# Anomalous Paramagnetic Meissner-like AC Response in EuRbFe_4_As_4_ Superconductor

**DOI:** 10.3390/ma19071365

**Published:** 2026-03-30

**Authors:** Adrian Crisan, Alina M. Badea, Ion Ivan, Corneliu F. Miclea, Daniel N. Crisan, Armando Galluzzi, Massimiliano Polichetti

**Affiliations:** 1 National Institute of Materials Physics, 405A Atomistilor Str., 077125 Magurele, Romania; alina.ionescu@infim.ro (A.M.B.); ion.ivan@infim.ro (I.I.); miclea@infim.ro (C.F.M.); daniel.crisan@infim.ro (D.N.C.); 2 Department of Physics “E.R. Caianiello”, University of Salerno, Via Giovanni Paolo II, 132, 84084 Fisciano, Italy; agalluzzi@unisa.it (A.G.); mpolichetti@unisa.it (M.P.)

**Keywords:** magnetic superconductor, EuRbFe_4_As_4_, AC susceptibility, paramagnetic Meissner-like response

## Abstract

Magnetic superconductor EuRbFe_4_As_4_ is a quite unique system in which macroscopic superconductivity and magnetic ordering coexist, with interesting interactions between Abrikosov vortices and Eu^2+^ spins that were investigated mostly by static (DC) magnetization measurements. Our aim is to study the dynamic interactions between the two sub-systems using AC susceptibility measurements in a wide range of temperatures and superimposed DC fields. In low DC fields, the magnetic transition at 15 K is clearly visible. We have observed very little difference between the AC susceptibility in different cooling regimes, but large difference for different field orientation. For field perpendicular to the superconducting planes, we have observed an anomalous dependence just below the critical temperature, which is absent in the parallel field orientation. We explained the anomaly by the interplay between the sample dimensions and the temperature dependence of the London penetration depth which may allow the paramagnetic Meissner-like response to be detected in the temperature dependence of the AC susceptibility. We stress that the newly reported phenomenon reflects an AC-susceptibility manifestation of a field-stabilized critical state rather than a thermodynamic phase. In addition, we have observed a paramagnetic AC response in the normal phase, in both field orientations, indicative of interactions between Eu^2+^ spins and flux lines.

## 1. Introduction

**Figure 1:**
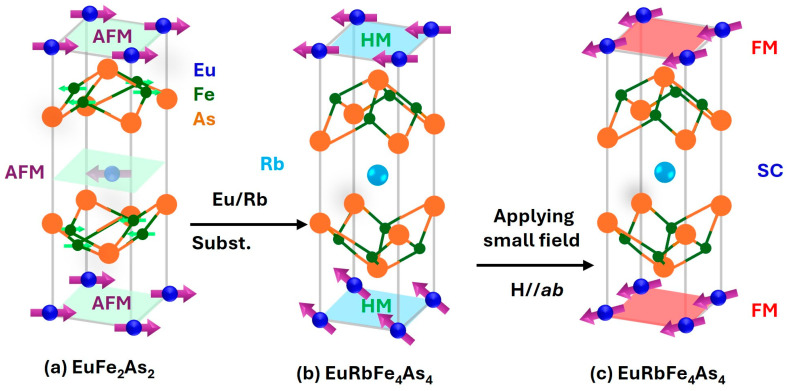
Schematic structures of: (**a**) EuFe_2_As_2_; (**b**) EuRbFe_4_As_4_ with helical magnetism of Eu^2+^ spins; and (**c**) EuRbFe_4_As_4_ with ferromagnetism of Eu^2+^ spins induced by magnetic field.

Soon after the discovery of iron-based superconductors (IBSs) [1], the 122 family based on *AE*Fe_2_As_2_ (*AE* being alkali-earth metal Ca, Sr, Ba) parent compound became the most popular material for both physical explorations and applications because of its critical temperature *T*_c_ as high as 38 K [2], very high upper critical fields µ_0_*H*_c2_ (>70 T) [3,4] and low anisotropies (γ < 2) [4]. Superconductivity in *AE*Fe_2_As_2_ is primarily induced by alkali metal (*A* = Na, K, Rb, Cs) substitution at *AE* sites with a concomitant suppression or elimination of the structural and magnetic ordering transition. The structure’s crystallographic space group (*I4/mmm*) is not changed by this *A* substitution because *AE* and *A* randomly occupy crystallographically equivalent sites. Thus, (*AE*_1−x_*A*_x_)Fe_2_As_2_ (also noted as (*AE*,*A*)Fe_2_As_2_) are solid solutions between *AE*Fe_2_As_2_ and *A*Fe_2_As_2_ compounds with the same structural type. Later it was found that, if there is a large difference in the ionic radii Δ*r* of *AE* and *A*, such solid solutions are not possible and a new type of IBSs has been reported [5], having a new structure, abbreviated as *AEA*1144, namely Ca*A*Fe_4_As_4_ (*A* = K, Rb, Cs) and Sr*A*Fe_4_As_4_ (*A* = Rb, Cs). In these cases, because *A* does not mix with *AE* due to the large Δ*r*, *AEA*1144 crystallizes through alternate stacking of the *AE* and *A* layers across the Fe_2_As_2_ layers, changing the space group from *I4/mmm* to *P/4 mmm*, the compounds being superconductors with *T*_c_ values between 31 and 36 K. One of the most studied superconductors (SCs) in this new family is CaKFe_4_As_4_ (CaK1144) due to its excellent superconducting properties including very high critical current density and very high pinning potential [6]. Following the discovery of the 1144-type IBS, in 2016 two Eu-containing materials in the family, *A*EuFe_4_As_4_ (*A* = Rb, Cs), were subsequently synthesized and characterized [7,8]. The two sibling compounds exhibit SC at *T*_c_ = 36.5 K (*A* = Rb) and 35 K (*A* = Cs), respectively, without extrinsic doping. The Eu^2+^ spins order at *T*_m_ = 15 K (*A* = Rb) or 15.5 K (*A* = Cs) [8]. The Mössbauer studies [9] indicate that the Eu^2+^ spins in *A*EuFe_4_As_4_ are ferromagnetically (FM) coupled and lie flat in the *ab* plane. Intuitively, the removal of every alternating magnetic Eu layer in EuFe_2_As_2_ would give rise to Eu-spin FM. Nevertheless, recent neutron diffraction study [10] revealed a helical modulation with the magnetic propagation vector of **k** = (0, 0, 1/4) for the Eu-spin ordering. If a small magnetic field (∼0.2 T and ∼0.4 T, respectively, for *H // ab* and *H // c*) is applied [11], this helically modulated magnetic structure easily changes into a genuine FM, the latter of which fully coexists with SC. Therefore, the new materials can be viewed as a natural atomic-thick superconductor–ferromagnet superlattice, as sketched in Figure 1. The parent compound EuFe_2_As_2_ is an antiferromagnet (Figure 1a). Due to the large difference in atomic radii, a 50% substitution of Eu with Rb results not in (Eu_0.5_Rb_0.5_)Fe_2_As_2_ solid solution, but in the new, layered 1144 structure, EuRbFe_4_As_4_, in which the Eu layers having helical magnetism sandwiches a thick superconducting layer composed of two Fe_2_As_2_ planes with an Rb plane between them (Figure 1b). By applying a small magnetic field, helical magnetism in the Eu planes is driven into a ferromagnetic state (Figure 1c).

The new magnetic superconductor was characterized by many techniques to elucidate various aspects of superconductivity. After their discovery by Kawashima et al. [7], the crystal structure was investigated by XRD and determined to be tetragonal with symmetry group P4/*mmm* and by resistivity and DC magnetization that showed a critical temperature of about 36 K, an upper critical field (extrapolated at 0 K) of about 92 T and a coherence length of 1.8 nm [7]. More importantly, they discovered an anomaly in the magnetic response at 15 K which was correctly interpreted as coexistence of superconductivity and a magnetic ordered state created by Eu^2+^ ions. A few months later, Liu et al. [8] managed to replicate the material, and, in addition to resistivity and magnetization measurements that confirmed previous results, they also performed magnetization hysteresis measurements and specific heat measurements that revealed a very rare third-order type magnetic transition. In a seminal paper, Ishida et al. [12], by combining neutron diffraction and magnetization measurements, revealed that ferromagnetic alignment of Eu^2+^ moments is induced by superconducting vortices. They showed that the direction of the Eu^2+^ spins is dominated by the distribution of pinned vortices based on the critical state model, highlighting a unique interplay between magnetism and superconductivity. Vortex matter, dynamics and pinning are reflected differently in the case of AC susceptibility as compared to DC studies. For this reason, in this work we investigated the interplay between magnetic moments and vortex matter and dynamics in the case of AC fields superimposed on DC fields up to 9 T, for fields orientations perpendicular and, respectively, parallel to the superconducting planes, in both zero-field cooling (ZFC) and field cooling (FC) procedures. The results showed the expected ferromagnetic signal at around 15 K superimposed on the diamagnetic screening at low DC fields (similar to DC susceptibility measurements), as well as a clear anomaly in the diamagnetic screening due to ferromagnetic ordering of the spins immediately after *T*_c_ at higher fields. The anomaly in the in-phase susceptibility is accompanied by a shoulder in the out-of-phase susceptibility (dissipation peak), in both ZFC and FC regimes, for perpendicular orientation. In the case of measurements with the thin sample parallel to the fields, such anomalies were not detected. 

## 2. Materials and Methods

The EuRbFe_4_As_4_ single crystals were grown in AIST Tsukuba, Japan, by the RbAs-flux method [13], in which EuAs, Fe_2_As and RbAs precursors were prepared from Eu and As, Fe and As, and Rb and As, respectively, which were thoroughly mixed at appropriate molar ratios. EuAs and Fe_2_As mixtures were sealed in evacuated quartz tubes, while RbAs was sealed in a stainless-steel tube within an alumina crucible, followed by sintering processes (750 °C for EuAs, 900 °C for Fe_2_As and 600 °C for RbAs). The sintered powders were weighted in the ratio 1:1:15 to a total amount of 9g and placed in an alumina crucible, sealed in a stainless-steel container. The thermodynamical process of crystal growth consisted in heating the sample to 700 °C in 5 h and maintaining this temperature for 5 h; then, it was heated to 970 °C in 5 h and this temperature was maintained for 10 h, with a final step being very slow cooling for 350 h (1 °C/h) to 620 °C. After cleaving the surfaces of the single crystals, XRD patterns showed only the (00*l*) peaks from EuRbFe_4_As_4_ [12]. The sample investigated in this work is a thin square with length *l* = 0.9 ± 0.1 mm and thickness *t* ≈ 0.06 ± 0.02 mm.

AC magnetic susceptibility was measured using a *Quantum Design* PPMS-9 T (Quantum Design Inc., San Diego, CA, USA) with an ACMS option and the PPMS MultiVu^TM^ 1.5 software (Quantum Design), which allows to pre-program the type of measurements, the temperature (fixed or variable, between 1.9 and 350 K), the field (fixed or variable, up to 9 T), the frequency of the AC field excitation (up to 10 kHz), and the amplitude of the AC field excitation (up to 16 Oe). For this work, AC susceptibility measurements were conducted as a function of temperature by applying an AC magnetic field perpendicular or parallel to the superconducting layers (*a*–*b* planes), with or without a superimposed DC magnetic field up to 9 T, in the zero-field cooling (ZFC) conditions or field cooling (FC) conditions. Most of the measurements were taken at a fixed AC field amplitude *h*_AC_ = 1 Oe and at the AC field frequency ν = 5686.4 Hz. For a qualitative estimation of the influence of the AC frequency on the observed anomaly, we have repeated the measurements for two more frequencies, 497 and 9997 Hz, and with a larger AC field amplitude of 10 Oe. To prevent any potential effects of residual field trapped within the DC magnet on the sample response, before each measurement we warmed the magnet close to room temperature, applied 2 T and reduced the field to zero in the oscillation mode. This demagnetizing process was proven to reduce the trapped DC field below 1 Oe. After demagnetizing procedure, the sample was cooled to 5 K in ZFC or FC conditions. Susceptibility as function of temperature data were measured between 5 and 40 K with a sweep rate of 0.1 K/min, such small sweeping rate ensuring a large number of measured points for each curve.

## 3. Results

### 3.1. Fields Perpendicular to the Superconducting Planes

**Figure 2:**
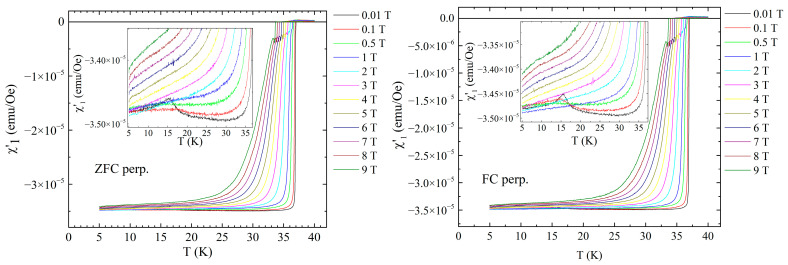
Temperature dependence of the in-phase susceptibility for the DC fields indicated in the figure, for fields perpendicular to the superconducting planes, in ZFC (**left-hand side**) and FC (**right-hand side**) cooling protocols. Inserts show details of the diamagnetic signal around the temperature of the magnetic transition (15 K).

Figure 2 shows the temperature dependence of the in-phase fundamental susceptibility of the sample measured with an AC field with amplitude of 1 Oe and frequency of 5686.4 Hz superimposed on DC magnetic fields of 0.01, 0.1, 0.5, 1, 2, 3, 4, 5, 6, 7, 8 and 9 T.

**Figure 3:**
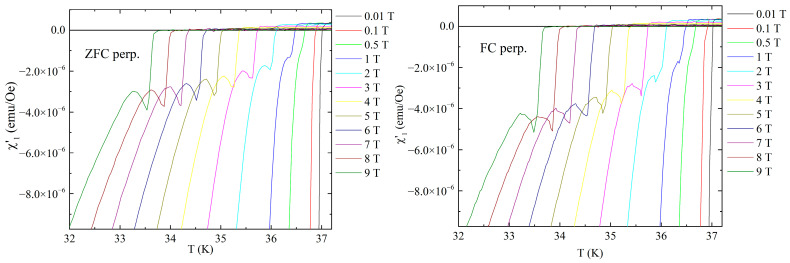
Temperature dependence of the in-phase susceptibility in the perpendicular configuration, in zero-field cooling (**left-hand side**), and field cooling (**right-hand side**) very close to the critical temperatures (zoom of the anomaly from Figure 2).

The first thing we observed is the small signal at low temperature and very low DC fields (<0.5 T) that indicates the magnetic transition, similar to the ones detected in DC magnetization measurements [7,8]. In higher fields the ferromagnetic signal from Eu^2+^ is masked by the decrease in diamagnetic screening due to the decrease in *J*_c_ with increasing DC field. Near *T_c_* we observe a standard sharp superconducting transition in low DC fields, which, as expected, becomes wider and has smaller on-set temperature with increasing DC fields. However, for DC fields higher than 0.5 T the in-phase susceptibility shows an unexpected anomaly not far from *T*_c_ as can be seen in the main panels and, in detail, in Figure 3. After the normal increase in diamagnetic screening with decreasing temperature, we can see a decrease in diamagnetic screening, which is consistent with the appearance of a small ferromagnetic signal from the Eu^2+^ spins. The anomaly starts as a “shoulder” for fields smaller than about 2 T and develops as a clear local “maximum” for larger DC fields. Looking at both sets of graphs, we can see that there are small, but not significant differences between ZFC and FC protocols.

**Figure 4:**
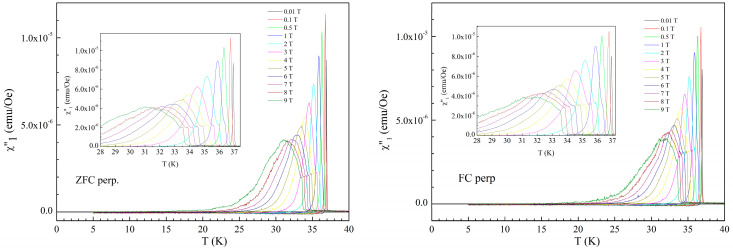
Temperature dependence of the out-of-phase susceptibility for the DC fields indicated in the figure, for fields perpendicular to the superconducting planes, in ZFC (**left-hand side**) and FC (**right-hand side**) cooling protocols. Inserts show a more detailed picture of the dissipation signal.

Figure 4 shows the temperature dependence of out-of-phase susceptibility, in both ZFC and FC protocols, for the same DC fields as in Figure 2 and Figure 3. Figures show again very small, negligible differences between ZFC and FC protocols. For small fields, the dissipation signal has a very sharp peak and, as DC fields exceed 0.5 T, a “shoulder” is developing and becomes more visible with the increase in DC field, together with the expected broadening of the dissipation signal. 

The positions (temperatures) of the shoulders correspond to those of the local “peaks” in the in-phase susceptibility as seen in Figure 2 and Figure 3.

**Figure 5:**
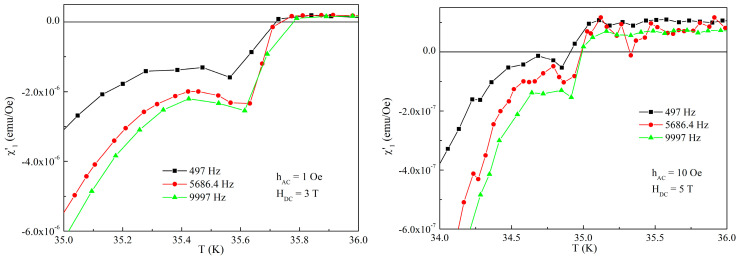
Temperature dependence of the in-phase susceptibility for the DC fields, AC fields amplitudes, and AC field frequencies indicated in the figure.

Since the AC susceptibility is inherently sensitive to both amplitude and frequency of the excitation AC field, we have performed additional experiments with two more frequencies, 497 and, respectively, 9997 Hz, as well as with AC field amplitude of 10 Oe in a few DC fields. Two such examples are provided in Figure 5. It can be seen that, although there are quantitative differences in the temperature dependence of the susceptibility in respect with different AC frequency and AC field amplitude, ***the anomaly is still there***. For a quantitative discussion on the influence of the AC excitation field amplitude and frequency on the anomalous paramagnetic Meissner-like response, a lot more data are required, which is not the aim of this paper. 

A closer look at the data in the normal state, for AC in-phase susceptibility for temperature above *T*_c_, reveals a positive AC susceptibility response. In principle, this can be attributed to a parasite signal from the sample holder and/or other artifacts in the measurement. To address this issue, we have revisited the measurements on the “brother” compound CaKFe_4_As_4_, which has the same structure (1144) but it does not have any atoms with spins. The single crystals were characterized in the same equipment, using similar procedures. Moreover, the single crystals of the two systems were grown in the same laboratory in AIST Tsukuba, Japan. In CaK1144, the data in the normal state are all in the noise level, at about ±10^−8^ emu/Oe, while in EuRb1144 there is a clear positive AC susceptibility response in the normal state, decreasing from 4.6 × 10^−7^ emu/Oe at 0.01 T to 4 × 10^−7^ emu/Oe at 0.1 T, then to 3 × 10^−7^ emu/Oe between 0.5 and 1 T, and, finally, to 2 × 10^−7^ emu/Oe at 3 T. At 5 T, we still have a positive susceptibility above the noise level of 8 × 10^−8^ emu/Oe, and, at 7 T, there is just the noise level of 10^−8^ emu/Oe. This comparison is a straightforward proof that the AC susceptibility measurements in EuRb1144 are able to probe the interaction between the Eu^2+^ spins, the DC magnetic flux lines in the sample in the normal state, and the AC field excitation. In low DC fields, it is easier for the AC field excitations to partially flip the Eu^2+^ spins, so the signal is larger than in the higher DC fields, where the interaction between the static high-density flux lines and the Eu^2+^ spins is stronger.

### 3.2. Fields Parallel with the Superconducting Planes

**Figure 6:**
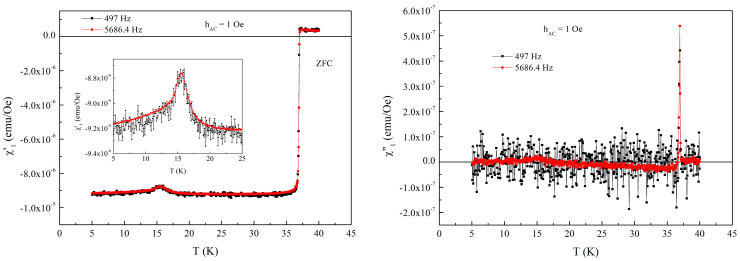
Temperature dependence of the in-phase (**left-hand side**) and out-of-phase (**right-hand-side**) susceptibility in zero DC field, with AC field amplitude of 1 Oe and the two AC frequencies indicated in the figure. Insert shows the detail of the signal due to magnetic transition at 15 K.

For this geometry, we have performed the measurements with two AC field amplitude, 1 and 10 Oe, and with two frequencies, 497 and 5686.4 Hz, in ZFC protocol. After looking at the results at DC fields 0 and 0.01 T, we have seen that the signal for 1 Oe and 497 Hz is very noisy, as can be seen in Figure 6. Even for the low frequency, we can see a sharp superconducting transition in both diamagnetic screening and dissipation peak. The magnetic transition is clearly visible at 15 K, similar to the case of perpendicular orientation and DC susceptibility measurements.

For AC field amplitude of 10 Oe the measurements look similar, only without the noise at low frequency. For DC field of 0.01 T (100 Oe), both ZFC and FC measurements with AC field amplitudes of 1 and 10 Oe look similar to the zero DC field case (again with quite large noise at 1 Oe and 497 Hz). The data can be seen in Supplementary Material Figure S1.

For higher fields, the measurements in parallel configuration show standard in-phase susceptibility, without any anomaly just below *T*_c_ as was the case of perpendicular configuration. To summarize the results of the measurements in parallel configuration in zero or very small DC fields: the superconducting transitions are sharp, with the expected magnetic signal in the in-phase susceptibility at 15 K, very similar to measurements in perpendicular configuration and to low-field DC measurements reported in the literature. In higher fields, there is no anomaly in the diamagnetic signal as is the case for perpendicular fields configuration. The small positive signal has the same values (DC field-dependent) as in the perpendicular configuration.

### 3.3. Comparison Between Perpendicular and Parallel Configurations

**Figure 7:**
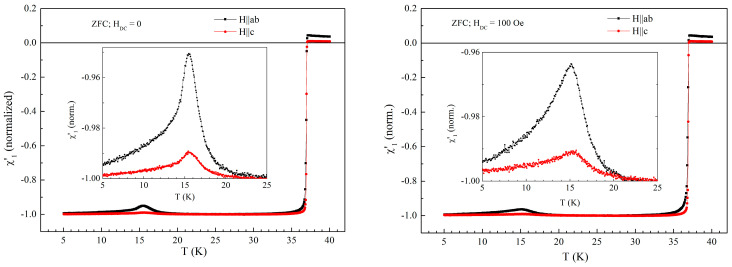
Temperature dependence of the in-phase susceptibility in zero DC field (**left-hand side**) and in DC field of 100 Oe (**right-hand-side**), measured with AC field amplitude of 1 Oe and AC frequency of 5686.4 Hz. Inserts show the details of the signal due to magnetic transition at 15 K.

Since we have not observed any anomaly in the in-phase susceptibility in parallel orientation, it is interesting to have a comparison between χ’_1_ (*T*) for the two orientations, in the same DC field, measured with the same amplitude and frequency of the AC field. For such comparison to be meaningful, we need to take into account the huge difference in demagnetization factors for the two field orientations due to the plate-like shape of the sample with thickness one order of magnitude smaller than the larger dimensions, so we normalized the in-phase susceptibility curves to the largest values of the diamagnetic signal for the two orientations, which are about −3.5 × 10^−5^ emu/Oe in perpendicular configuration (see Figure 2), and, respectively, about −0.9 × 10^−6^ emu/Oe in parallel configuration (see left-hand-side of Figure 6), this way ensuring in both orientations the in-phase normalized susceptibility at 5 K is −1 (perfect diamagnetism). Without normalization, the signal in the parallel configuration will be very small in comparison with perpendicular configuration, any detail being almost invisible. All normalized susceptibility curves shown below were measured in various DC fields using ZFC protocol, with AC field amplitude of 1 Oe and frequency of 5686.4 Hz. FC protocols gave the same results, with small, minor differences. Figure 7 shows the results for zero and 100 Oe, below the transition between helical and ferromagnetic arrangements of the Eu^2+^ spins. It can be seen that, in zero and very low DC field, the superconducting transitions are sharp, with the same *T*_c_. The signal due to magnetic transition is 5 times larger in the case of parallel orientation, which can be easily explained considering that it is much easier for the Eu^2+^ spins to follow the direction of the AC field in the *a*–*b* planes for parallel orientation than to tilt away from the planes to follow the direction of perpendicular AC field. The absolute value of positive susceptibility in the normal state is roughly the same, for the same DC field, in both field configurations. This is easy to explain, because in the normal phase there is no demagnetization factor to take into account. Remarkably, the value of the positive susceptibility in the normal state for parallel field is about the same as the height of the peak at 5 K, namely about 0.05 a.u. on the normalized scale. A larger signal for the parallel orientation at the magnetic transition at 15 K was observed also in DC magnetization measurements in a DC field of 30 Oe [14].

**Figure 8:**
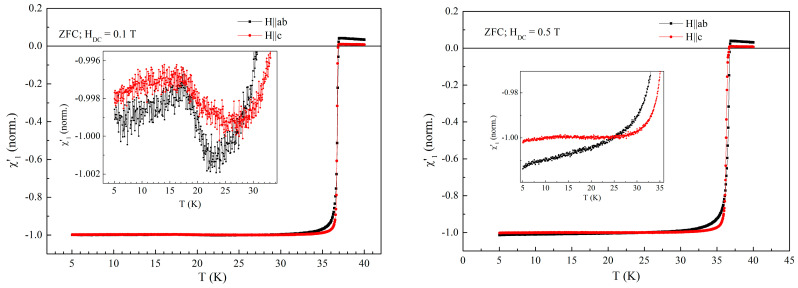
Temperature dependence of the in-phase susceptibility in DC field of 0.1 T (**left-hand side**) and in DC field of 0.5 T (**right-hand-side**), measured with AC field amplitude of 1 Oe and AC frequency of 5686.4 Hz. Inserts show the details of the signal due to magnetic transition at 15 K.

Figure 8 shows the temperature dependence of the in-phase fundamental susceptibility for the two orientations, for fields above the field of helical magnetism/ferromagnetism transition, but not very large, 0.1 T (left-hand side) and 0.5 T (right-hand side). It can be seen that, in such intermediate fields, there is a competition between the magnetic interaction of Eu^2^+ spins with the larger density of vortices induced by the DC field (similar to the so called “magnetic pinning”) and the tendency to follow the AC field orientation. In 0.1 T the signals due to magnetic transition are much smaller and very noisy, while in 0.5 T the signal practically disappears.

**Figure 9:**
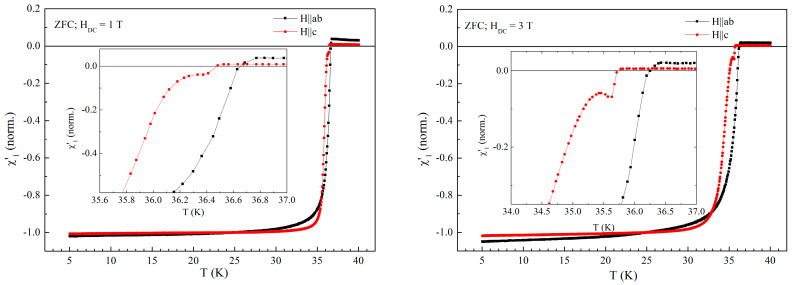
Temperature dependence of the in-phase susceptibility DC field of 1 T (**left-hand-side**) and in DC field of 3 T (**right-hand-side**), measured with AC field amplitude of 1 Oe and AC frequency of 5686.4 Hz. Inserts show the details of the transition just below *T*_c_.

In higher fields there is no more visible signal at 15 K due to the much larger density of vortices which, through magnetic interaction, impede the Eu^2+^ spins from following the direction of AC field, but for such larger DC fields the anomaly just below *T*_c_ in perpendicular configuration appears. Figure 9 shows the comparison between the temperature dependence of the normalized in-phase susceptibility for parallel and perpendicular configurations in DC fields of 1 T (left-hand-side) and 3 T (right-hand-side). Inserts show details of curves just below *T*_c_ where the anomaly starts to appear for perpendicular configuration. Apart from the anomaly, there are a few interesting features. The most visible is the appearance of a small difference in the critical temperatures for the two orientations, due to the large demagnetization factor in perpendicular configuration. Another feature is the decrease in the positive signal in the normal state with increasing DC field, again due to the magnetic interaction between Eu^2+^ spins and magnetic flux lines with an increasing density, preventing the spins to follow the direction of AC field. It is worth noting that we have kept the same normalizing factors as in zero DC field; this is the reason for in-phase susceptibility for parallel orientation at low temperature laying below −1. We should also note that the actual positive susceptibility in the two directions is the same, between 2 × 10^−7^ and 4 × 10^−7^ emu/Oe depending on the DC field, which is to be expected since in the normal phase there is no demagnetization effect. So, the apparent difference in the signal at higher temperatures in Figure 9 is just an artifact due to the normalization procedure of the signal in superconducting state.

## 4. Discussion

We will start our discussion with a comparison between the EuRb1144 magnetic superconductor and its more studied “relative” non-magnetic CaK1144. Quite unexpectedly, EuRb1144 showed a higher *T*_c_ of 37 K as compared to 35.8 K in CaK1144 [6,15] despite the presence of magnetism which usually suppresses critical temperature. However, the interaction between Eu^2+^ spins and the vortex system leads to a much broader superconducting transition in high magnetic fields and a more pronounced reduction in *T*_c_ with increasing DC field. For example, in a DC field of 9 T, *T*_c_ decreases with 10% in EuRb1144 (see Figure 3) while in CaK1144 *T*_c_ decreases with only 8% (see Supplementary Material Figure S2). Another important aspect is the comparison between ZFC and FC conditions. Similar to CaK1144, there is no significant difference between ZFC and FC AC susceptibility in EuRb1144, unlike the case of isovalently substituted BaFe_2_(As_1−x_P_x_)_2_ 122 compound which showed a very pronounced magnetic memory effect [16,17]. Of course, the main difference between the two 1144 materials is the signal around 15 K due to the magnetic transition, which was first detected through DC magnetization measurements and reported in the same papers that announced the new material [7,8]. Also, the existence of a clear ***positive AC susceptibility in the normal state***, which is DC field-dependent, due to the interaction between the Eu^2+^ spins and the superimposed DC and AC fields, is quite remarkable. For comparison, the AC susceptibility in the CaK1144 system is zero (within the noise level, ±10^−8^ emu/Oe). Another feature that was not observed previously in any studies is the ***anomaly in the susceptibility response to the AC field perturbation just below T_c_*** in fields larger than approximately 0.5 T, ***only with the fields perpendicular to the ab planes***. We propose that the explanation of this anomaly is due to the interplay between the temperature dependence of the London penetration depth, λ*_ab_*, near *T*_c_, and the dimensions of the sample, in the context of the very peculiar interaction between the vortex system and the subsystem of Eu^2+^ spins [18,19]. Vlasko-Vlasov et al. [18] performed a study of the magnetic-flux evolution in EuRb1144 using magneto-optical imaging and DC magnetization measurements. They showed that the interplay of magnetic susceptibility amplifying magnetic induction and vortex pinning attenuating the magnetic flux entry results in a field- and temperature-dependent critical state that emulates a paramagnetic Meissner effect. They further concluded that the observed vortex dynamics corresponds to a nontrivial spatial current distribution and yields a self-consistent inhomogeneous enhancement of the sample magnetization. Suppression of superconductivity by correlated magnetic fluctuations was also detected by high-resolution scanning Hall probe microscopy [19]. Our results are different from the Wohlleben effect (paramagnetic Meissner effect), in which, for certain high-*T*_c_ superconductors (e.g., Bi2212 and Bi2223), they exhibit a paramagnetic response when cooled in a low magnetic field. This effect has been attributed to trapped magnetic flux or π-junctions in granular superconductors that can create spontaneous, persistent currents, at very low constant magnetic fields < 1 Oe. A comprehensive review of Wohlleben paramagnetic Meissner effect can be seen in [20]. In our case we are dealing with single crystals (not granular superconductors), high magnetic fields, AC susceptibility response in both ZFC and FC conditions. To explain the origin of the anomalous susceptibility response in perpendicular configuration we are extending the concept of paramagnetic Meissner effect (*not Meissner phase as thermodynamic concept*) described in [18] to the case of penetration of AC perturbation of the mixed state stabilized by a large DC field. In this context, just below *T*_c_ the superimposed AC magnetic field disturbs the critical state inside the sample on a scale of London penetration depth, λ*_ab_*, resulting in the AC susceptibility response of the sample. We would describe the region of the sample not perturbed by the AC excitation (in the center of the sample) as paramagnetic Meissner-like state following [18], although it contains Abrikosov vortices (not perturbed by AC magnetic field excitation) as well as oriented Eu^2+^ spins.

**Figure 10:**
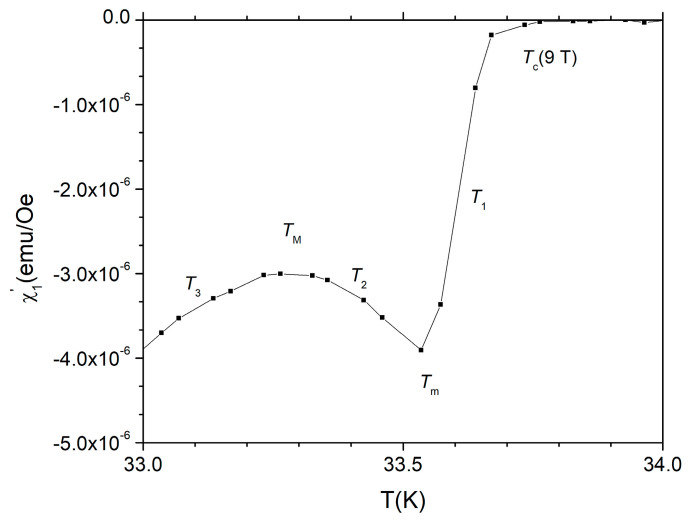
Details of temperature dependence of in-phase susceptibility just below *T*_c_ for *H*_DC_ = 9 T.

For a clearer *qualitative* explanation of the anomaly just below *T*_c_ we are using the experimental data (detail near the superconducting transition) of the temperature dependence of the in-phase susceptibility χ’ in a DC field of 9 T, showed in Figure 10. The temperatures indicated in the figure are *T_c_* (9 T) = 35.8 K; *T*_1_ is a temperature in the first part of increasing diamagnetic signal, *T*_m_ is the temperature of the starting of anomalous susceptibility response, *T*_2_ is a temperature where the diamagnetic response decrease with decreasing temperature due to Eu^2+^ spins, *T*_M_ is the temperature where the derivative *dχ’*/*dT* changes sign again and *T*_3_ a temperature at which the circulating AC currents results in diamagnetic AC shielding overcoming the paramagnetic Eu^2+^ spins paramagnetic response due to much higher critical current density at this lower temperature, with the trend continuing towards lower temperature in the expected way.

As previously mentioned we suggest that the anomalous Meissner-like AC susceptibility very near *T*_c_(*H*_DC_) is determined by the interplay of sample dimensions, the interactions between Eu^2+^ spins and magnetic fields, and the temperature dependence of the penetration depth. In a previous work, we have investigated the vortex melting lines (which, for superconductors with small anisotropy, is near the *H*_c2_ (*T*) line) in superconducting single crystals of 1144 and 122 iron-based superconductors [21]. The experimental data were very well described by a λ*_ab_*(*T*) given by the 3D *X-Y* critical fluctuations model, λ*_ab_*(*T*) = λ*_ab_*(0)(1 − *T/T*_c_)^−1/3^. However, in the case of the anomalous Meissner-like response, we are on the other side of the phase diagram, namely close to *H*_c1_ (*T*). In addition, the temperature dependences of London penetration depth in various models discussed in [21], i.e., 3D *X-Y* critical fluctuations, bi-fluid model, and mean-field model, were originated from well-known, classical adiabatic phonon-mediated superconductivity. 

In our case, these materials may be described within atypical non-adiabatic phonon-mediated framework [22,23], which seems also to be the case for some bismuthates [24]. Being discovered much earlier than EuRb1144, the first member of the 1144 family that was thoroughly investigated is CaK1144, using muon spin relaxation (µSR) [25], inelastic neutron scattering [26], µSR and angle-resolved photoemission spectroscopy (ARPES) [27], London penetration depth and tunneling conductance [28], ^75^As nuclear magnetic resonance (NMR) [29], all the experimental results being consistent with a two-gap *s*_+_ *s*_-_ wave model. It was also shown that the *H*_c2_ (*T*) dependence can be described by a two-band model in the clean limit with band-coupling parameters favoring intraband over interband interactions [30]. However, it seems that the band structure of CaK1144, and, in extension, of other materials in the 1144 family, including EuRb1144, is not that simple. A measurement of the anisotropy of the London penetration depth λ_L_ using a microwave-coplanar-resonator technique that allowed to de-convolute the anisotropic contributions λ_L,*ab*_ and λ_L,*c*_ resulted in a temperature dependence of the anisotropy parameter γ_L_ = λ_L,*c*_/λ_L,*ab*_ consistent with *ab initio* density-functional-theory (DFT) calculations showing that the Fermi surface consists of five bands centered around Γ with a hole character and three bands centered around *M* with an electron character [31]. It is obvious that the mechanisms of superconductivity in such complex structures as 1144 (even without additional Eu^2+^ spins as in the case of our sample) are not a closed subject; so, a *quantitative* explanation of the anomalous Meissner-like AC response, with DC and AC fields, AC field frequency, cooling procedures, field orientation, etc., as parameters, is out of reach, at least for us at this stage. 

For a *qualitative* explanation, we need to take into account that the experimental data, the sample geometry, and the temperature dependence of the London penetration depth are certainly not as straightforward as in classical superconductors. There are various experiments on CaK1144 dealing with temperature dependence of penetration depth, but the focus is on lower temperatures. We could not find in the literature data on λ_L_ (*T*) in EuRb1144 (or in CaK1144) for temperatures close to *T*_c_, but, since the superfluid density vanishes approaching *T*_c^’^_, the penetration depth will diverge, either following GL theory or other formula considering the very complex system. In addition, Eu^2+^ spins can modify λ_L_ (*T*) dependence.

**Figure 11:**
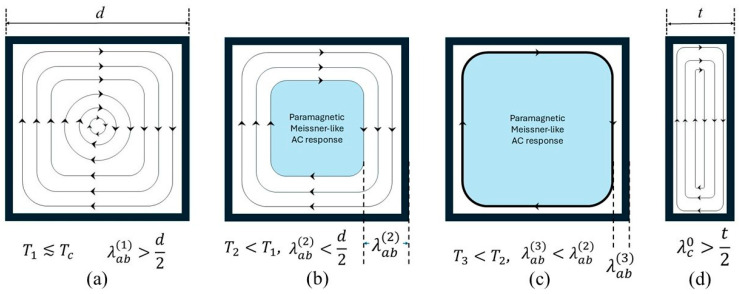
(**a**–**c**) Schematics of the circulating supercurrents in perpendicular orientation for three decreasing temperatures, same as those indicated in Figure 8, and (**d**) in parallel orientation.

Figure 11 is a sketch that helps understand our qualitative explanation for the anomaly in the AC susceptibility in EuRb1144, only in the perpendicular configuration. Figure 11a describes the penetration of the circulating AC supercurrents induced by the AC field excitation at temperature *T*_1_ indicated in Figure 10. At this temperature, λ*_ab_*(*T*_1_) is larger than the half-width of the sample, so the sample is fully penetrated by the AC perturbation, with a very small critical current density due to high temperature. Hence, there is the expected increase in the diamagnetic shielding due to the increase in critical current density with decreasing temperature, down to the temperature *T*_m_. 

With further decrease in temperature, as in Figure 11b, at *T*_2_, λ*_ab_*(*T*_2_) < *d*/2, the AC excitation does not penetrate all the sample, so in the middle there is a region with the paramagnetic Meissner-like AC response, region whose dimensions expand due to the further decrease in London penetration depth with decreasing temperature. Since the circulating supercurrent is still small due to closeness to *T*_c_, the paramagnetic signal from Eu^2+^ spins increases with decreasing λ*_ab_*(*T*) faster than the increase in critical current density, their combination leading to the anomalous decrease in the diamagnetic shielding between *T*_m_ and *T*_M_ as shown in Figure 10. Again, please note that the paramagnetic Meissner-like region is not a true Meissner phase as in the phase diagrams of type 2 superconductors, it indicates just the central region where the AC field perturbation does not penetrate. Further decrease in temperature below *T*_M_, at *T*_3_ (Figure 11c) lead to a larger paramagnetic Meissner-like region, but now the critical current density becomes large enough for the usual dependence of the diamagnetic shielding. 

Figure 11d explains the reason for the absence of the anomaly in the parallel configuration: the London penetration depth is much larger than the thickness of the crystal at temperatures close to *T*_c_, where the critical current is small enough. With decreasing temperature, penetration depth will decrease, which would lead to appearance of a region in the center of sample in which AC excitation does not penetrate, but at this temperature the critical (shielding) current is strong enough, so the diamagnetic response is much larger than the positive AC susceptibility due to Eu^2+^ spins. 

**Figure 12:**
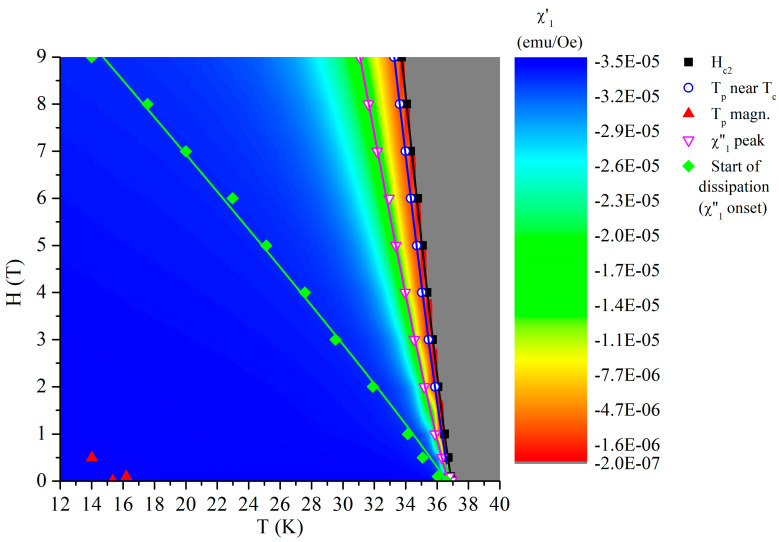
Schematic *H-T* diagram collecting superconducting and magnetic data/boundaries. The colored scale of the in-phase susceptibility is shown in the right-hand-side of the main panel, with meaning of the symbols shown in the top-right corner.

Before the conclusions chapter, a graphical summary of the main findings is shown in Figure 12 which visualizes the results within a compact *H-T* diagram, in perpendicular configuration and ZFC regime.

Starting from lower temperature, red triangle in the lower-left corner indicates the position of the peaks due to magnetic ordering near 15 K; green rhombs indicate the starting of dissipation (on-set of the out-of-phase susceptibility), magenta open triangles indicate the position of the peaks of out-of-phase susceptibility, blue circles represent the peak of anomalous response due to the paramagnetic Meissner-like AC response, and, finally, black squares indicate the field-dependent critical temperatures. As previously mentioned, in normal state the interaction between Eu^2+^ spins and magnetic flux lines results in positive AC susceptibility. In CaK1144, the data in the normal state are all in the noise level, at about ±10^−8^ emu/Oe, while in EuRb1144 there is a clear positive AC susceptibility response in the normal state, decreasing from 4.6 × 10^−7^ emu/Oe at 0.01 T to 4 × 10^−7^ emu/Oe at 0.1 T, then to 3 × 10^−7^ emu/Oe between 0.5 and 1 T, and, finally, to 2 × 10^−7^ emu/Oe at 3 T. These data are not indicated in the diagram.

## 5. Conclusions

Using AC susceptibility measurements, we investigated the interplay between Eu^2+^ spins and vortex matter and dynamics in the magnetic superconducting single-crystal EuRbFe_4_As_4_ with AC fields superimposed on DC fields up to 9 T, for fields orientations perpendicular and, respectively, parallel to the superconducting planes, in both zero-field cooling (ZFC) and field cooling (FC) procedures. For DC fields smaller than 0.5 T, in-phase AC susceptibility reveals the magnetic transition at around 15 K which manifests as a small decrease in diamagnetic superconducting shielding (a small local maximum), as was observed also in DC studies. In higher fields the ferromagnetic signal from Eu^2+^ is masked by the decrease in diamagnetic screening due to the decrease in *J*_c_ with increasing DC field. The expected mark of the spin ordering was observed in both field orientations and in both cooling conditions, with small differences in position and height of the local peak. A completely new discovery is the presence of an anomaly in χ’ (*T*) just below *T*_c_ in both cooling procedures (ZFC and FC), but only in the perpendicular field configuration. Just after the superconducting transition, the diamagnetic signal due to screening supercurrent decreases, then increases again upon cooling, as expected. We explained this anomalous paramagnetic Meissner-like AC response through the interplay between the sample dimensions, temperature-dependent penetration depth, temperature (and DC field) critical current *J*_c_ and the paramagnetic signal from the Eu^2+^ spins. Just below *T*_c_, since the London penetration depth is larger than the sample dimension, the AC field perturbation penetrates the whole sample, and the diamagnetic signal increases with decreasing temperature due to the increase in *J*_c_. With further decrease in temperature and subsequent decrease in λ_L_, the AC field perturbation does not penetrate the whole sample, and, in the center, there appears a region with paramagnetic Meissner-like AC response, which leads to decrease in the diamagnetic signal. With further decrease in *T*, the increase in *J*_c_ lead to diamagnetic signal to overcome the paramagnetic signal of Eu^2+^ spins. So, the condition for the anomaly to appear at a given temperature, in various DC fields, is a London penetration depth smaller than half-sample dimension (so there is in center a region free from AC field excitation, and, at the same time, a low-enough critical current density so the diamagnetic signal is smaller than the paramagnetic signal from Eu^2+^ spins). This is also the reason for just small shoulders in χ’ (*T*) in low DC fields, where *J*_c_ is large, while with increasing fields (decreasing *J*_c_) the anomaly has a very clear peak. The anomaly does not appear in the parallel configuration for the simple reason of sample dimension being much smaller and a smaller London penetration required a smaller temperature, where the critical current is already too large to be overcome by the paramagnetic signal of the spins. The anomaly was observed also in several experiments with different AC fields and frequencies, although, as expected, χ’ (*T*) are slightly shifted. Finally, another remarkable finding in our study is the existence of a clear paramagnetic response in the normal state, in both field orientations, of more than one order of magnitude larger than the noise level (and the normal state response in related CaK1144) due to the interaction between the Eu^2+^ spins and the magnetic flux lines. 

## Data Availability

The original contributions presented in this study are included in the article/Supplementary Material. Further inquiries can be directed to the corresponding author.

## Supplementary Materials

The following supporting information can be downloaded at: https://www.mdpi.com/article/10.3390/ma19071365/s1, Figure S1: Susceptibility of EuRb1144 in 0.01 T in conditions indicated in the graphs; Figure S2: Susceptibility of CaK1144.

## Abbreviations

The following abbreviations are used in this manuscript:

IBS	Iron-based superconductors
*AE*	Alkali-earth metal
*A*	Alkali metal
*AEA*1144	AEAFe_4_As_4_
FM	Ferromagnet/Ferromagnetic
ZFC	Zero-field cooling
FC	Field cooling
DC	Direct current
AC	Alternate current
PPMS	Physical Properties Measurement System
ACMS	Alternate Current Measurement System
