# Supplementary material for: Anomalous Paramagnetic Meissner-like AC Response in EuRbFe4As4 Superconductor
Source: Materials (Basel). 2026 Mar 30;19(7):1365. doi: 10.3390/ma19071365 (PMC13073782; doi:10.3390/ma19071365)
Supplement: Supplementary file 1 [file materials-19-01365-s001.zip › materials-4044457-supplementary.pdf]

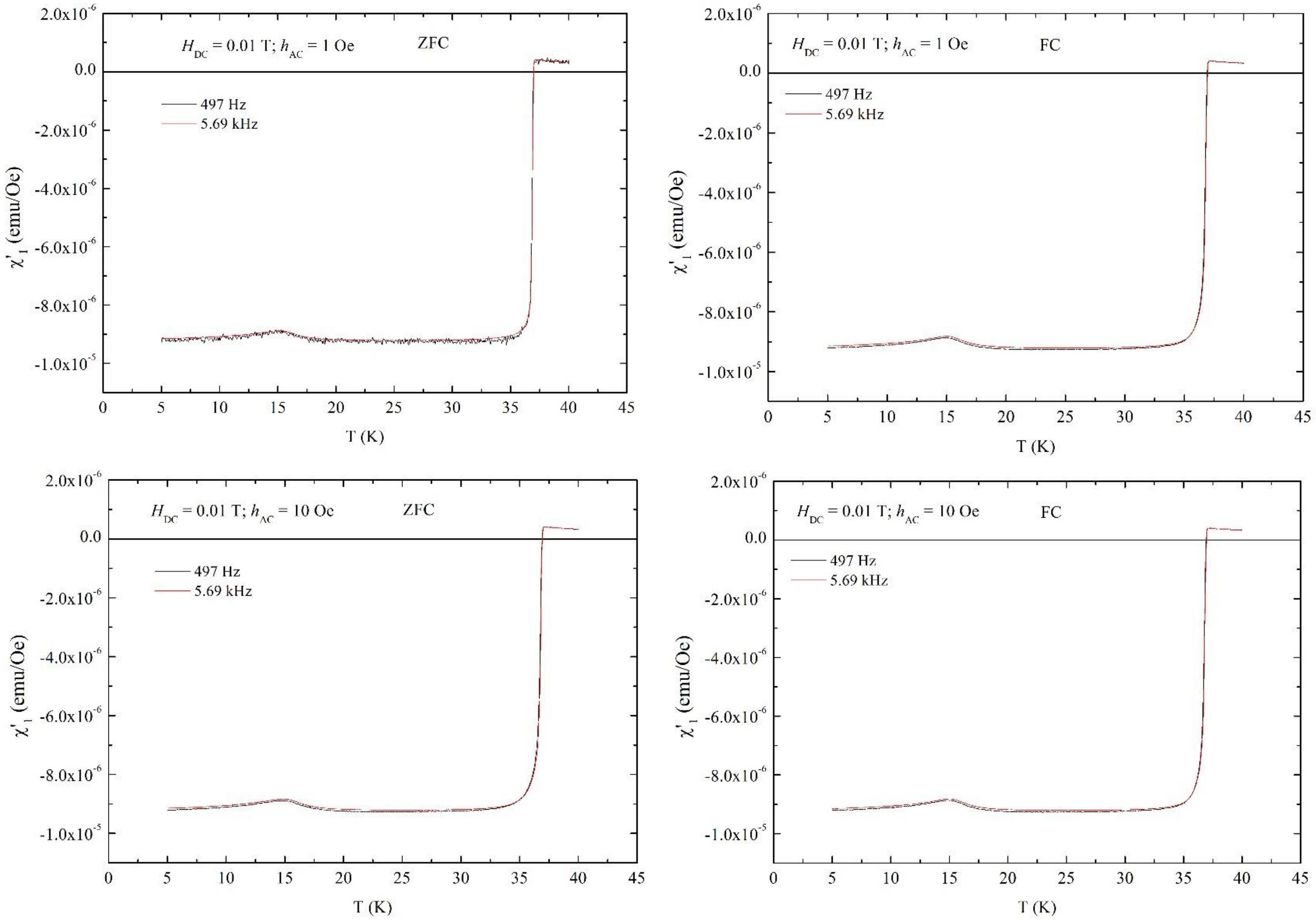

Supplementary Material Figure S1. Susceptibility of EuRb1144 in 0.01 T in conditions indicated in the graphs.

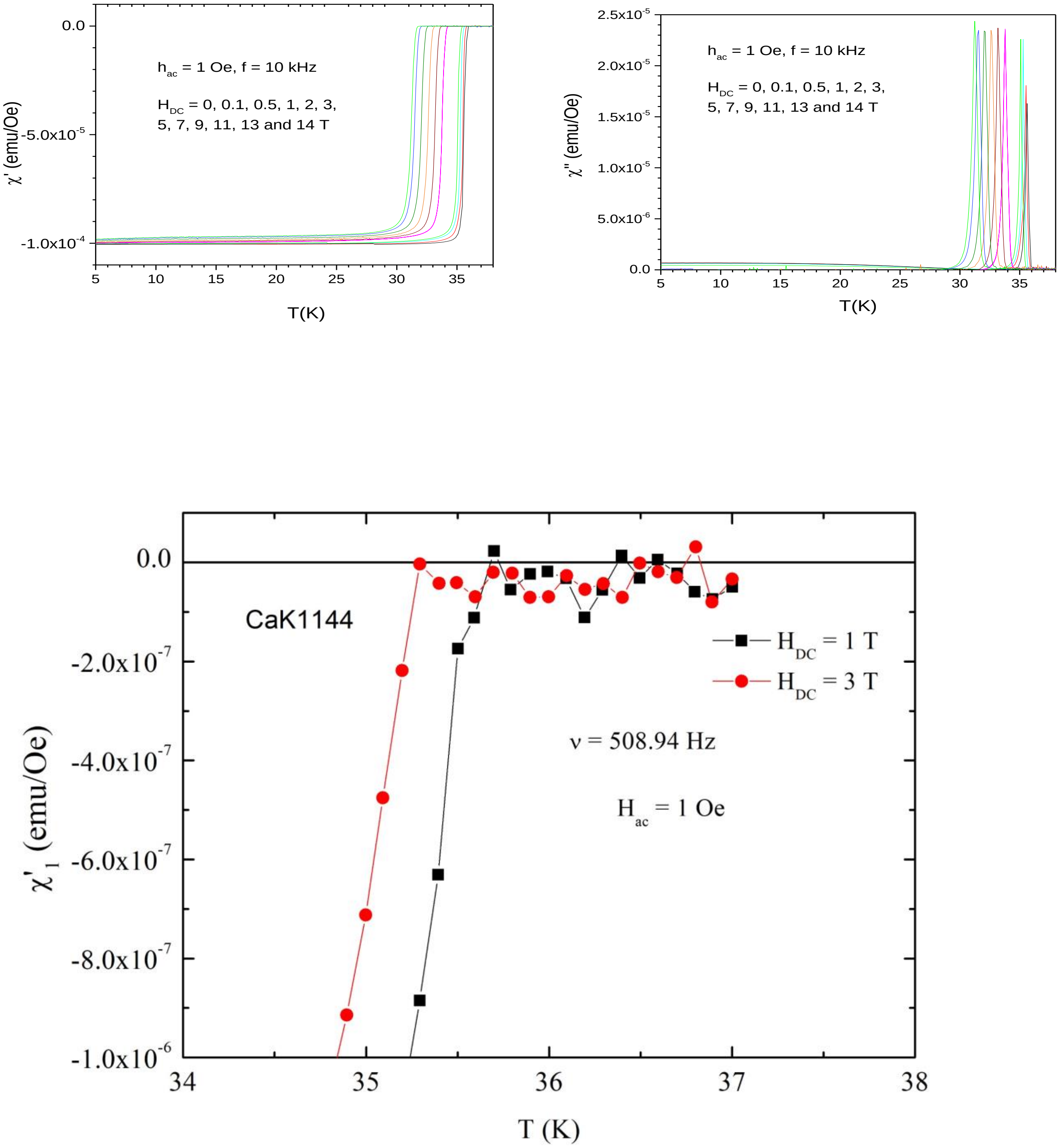

Supplementary Material Figure S2. Susceptibility of CaK1144
